# Impaired Cardiovascular Parameters in Resistance Training Practitioners Who Take Ergogenic Aids

**DOI:** 10.3390/jcdd10030113

**Published:** 2023-03-08

**Authors:** Bruno Bavaresco Gambassi, Daniela Conceição Gomes Gonçalves e Silva, Camila Almeida Sá, Roberto Rodrigues Bezerra, Cleilson Barbosa de Freitas, Marcelo Silva Costa, Paulo Roberto da Silva Marques, Pedro Paulo Ramos da Silva, Manoel Pereira Guimarães, Fabiano de Jesus Furtado Almeida, Richard Diego Leite, Dário Celestino Sobral Filho, Paulo Adriano Schwingel

**Affiliations:** 1 Laboratório de Pesquisas em Desempenho Humano (LAPEDH), Universidade de Pernambuco (UPE), Petrolina 56328-900, PE, Brazil; 2 Programa de Pós-Graduação em Gestão de Programas e Serviços de Saúde (PPGGPSS), CEUMA, São Luís 65075-120, MA, Brazil; 3 Departamento de Artes e Educação Física (DAEF), Universidade Estadual do Maranhão (UEMA), São Luís 65055-970, MA, Brazil; 4 Programa de Pós-Graduação em Ciências da Saúde (PPGCS), UPE, Recife 50100-130, PE, Brazil; 5 Laboratório de Força e Condicionamento, Centro de Educação Física e Desportos (CEFD), Universidade Federal do Espírito Santo (UFES), Vitória 29075-910, ES, Brazil

**Keywords:** autonomic nervous system, blood pressure, ergogenic resource, heart rate, sleep

## Abstract

Background: Although there are studies on blood pressure (BP) and autonomic cardiac control (ACC) impairments caused by ergogenic aids, research has scarcely addressed this analysis during sleep. This study analyzed BP and ACC during sleep and wake periods in three groups of resistance training (RT) practitioners: ergogenic aid non-users, thermogenic supplement (TS) self-users, and anabolic-androgenic steroid (AAS) self-users. Methods: RT practitioners were selected for the Control Group (CG; *n* = 15), TS self-users Group (TSG; *n* = 15), and AAS self-users Group (AASG; *n* = 15). All individuals underwent cardiovascular Holter monitoring (BP, ACC) during sleep and wake periods. Results: The maximum systolic BP (SBP) during sleep was higher in AASG (*p* < 0.01) than CG (*p* < 0.001). CG had lower mean diastolic BP (DBP) than TSG (*p* < 0.01) and lower mean SBP (*p* = 0.009) than the other groups. Additionally, CG had higher values (*p* < 0.01) than TSG and AASG for SDNN and pNN50 during sleep. HF, LF, and LF/HF ratio values during sleep were statistically different in CG (*p* < 0.001) from the other groups. Conclusions: Our findings demonstrate that high doses of TS and AAS can impair cardiovascular parameters during sleep in RT practitioners who take ergogenic aids.

## 1. Introduction

The increasing self-use of thermogenic supplements (TS) and anabolic-androgenic steroids (AAS) among young people who exercise regularly has been a concern among health professionals. Many resistance training (RT) practitioners try a variety of strategies to increase muscle mass and/or decrease body fat without solid scientific knowledge about such practices.

In this sense, Araújo, Andreolo, and Silva [1] observed that 34% of interviewed bodybuilders used supplements and 9% used AAS. Another piece of research demonstrated that 24% of interviewees used supplements, and 19% reported regular use of AAS [2]. Additionally, a study with 510 physically active people found that 76.1% of interviewees used supplements and/or AAS [3].

Given such a prevalence, it is crucial to verify the effects of TS and/or AAS self-medication on the risk of cardiovascular death. According to some studies, high TS doses cause negative changes in cardiovascular parameters [4,5,6]. In research conducted by Gomes Gonçalves e Silva et al. [7], significant differences in systolic BP (SBP) were observed comparing TS users with non-users. Additionally, abuse of AAS is also associated with cardiac arrhythmia, systemic arterial hypertension, cardiac hypertrophy, myocardial infarction, and sudden death [8,9,10,11,12,13,14,15,16,17].

Besides BP and ACC changes with a risk of cardiovascular death, there may also be an increased risk of complications depending on the period (e.g., sleep) when they are investigated. In this sense, O’Brien, Sheridan & O’Malley [18] have demonstrated that hypertensive patients with a lower nocturnal drop in BP had a higher prevalence of strokes, calling them “non-dippers”. In a recent study, Nakanishi et al. [19] observed that changes in nocturnal SBP may be associated with an increased risk of hypertensive brain injury. Zhang et al. [20] retrospectively assessed polysomnography data from 2111 volunteers and found worse ACC during sleep in patients with cardiovascular diseases than in individuals without them.

Although there are studies on BP and ACC impairments in ergogenic aid users, research has scarcely addressed this analysis during sleep. Given this and the health risks, the present study is necessary. Thus, the objective of this study was to analyze BP and ACC during sleep and wake periods in three groups of RT practitioners: ergogenic aid non-users, TS users, and AAS users.

## 2. Materials and Methods

### 2.1. Design

This analytical cross-sectional study was conducted upon approval by the Ethics Committee at the University of Pernambuco (CEP-UPE) and followed the ethical guidelines for human research according to the Declaration of Helsinki (1964, revised in 2013).

### 2.2. Participants

The sample comprised 346 physically active males, aged 18 to 50 years, recruited among RT practitioners at fitness centers in the Integrated Economic Development Region of Petrolina/PE and Juazeiro/BA in Brazil. Symptomatic volunteers with heart diseases (*n* = 33), diabetes (*n* = 11), competitive bodybuilders (*n* = 13), narcotic users (*n* = 43), smokers (*n* = 15), cosmetic doping self-users (*n* = 7), and excessive alcohol drinkers (*n* = 67) were excluded.

The initial sample (*n* = 157) comprised 15 RT practitioners using AAS during data collection without professional guidance (self-medication), 92 using TS without professional guidance (self-medication), and 50 ergogenic aid non-users. A total of 30 individuals were randomly selected among ergogenic aid non-users (control group [CG]; *n* = 15) and TS self-users group (TSG; *n* = 15) for comparison with the AAS self-users group (AASG; *n* = 15).

The CG comprised RT practitioners who reported not having ever used dietary supplements and/or AAS in their lives. TSG comprised regular users who had been taking, for at least 6 months, dietary supplements containing 1.3-dimethylamylamine (DMAA) or other ergogenic resources of the thermogenic type. AASG comprised users of testosterone and its synthetic derivatives. AASG participants did not have any medical prescriptions, and the substances were illegally acquired by users. Likewise, dietary supplements were used without any nutritional and/or medical prescription, and the compounds were purchased by users at registered stores. Participants were not offered any legal or illegal substances in this or any other phase of the research.

### 2.3. Assessments

#### 2.3.1. Anthropometric

Their height was measured with a portable scientific stadiometer (Seca, Hamburg, Germany) placed on the wall, with 0.1-cm accuracy. Total body mass was evaluated with a properly calibrated (ISO/IEC 17025: 2005) electromechanical scale W200/5 (Welmy Indústria e Comércio Ltda, Santa Barbara d’Oeste, SP, Brazil) with 50-g accuracy. Body mass index (BMI) was obtained by dividing the body weight in kilograms by the square of the body height in meters.

#### 2.3.2. Cardiovascular Parameters

BP and ACC were evaluated for 24 h with an electrocardiography Holter monitor and an ambulatory BP monitor (CardioMapa, Cardios, São Paulo, SP, Brazil). The most stable data in the 4-h sleep and wake period were used. Participants were instructed to maintain normal daily activities and record them in a personal diary.

R-R intervals (RRi) were recorded with a 3-channel ECG recorder (Cardiomapa, Cardios, São Paulo, SP, Brazil) at an 800-Hz sampling frequency in the supine position. After data collection, raw RRi data from ECG were exported in ASCII format to software supplied by the manufacturer.

Each participant’s skin was cleaned and prepared to fix surface electrodes (Red Dot^™^ 2560, 3M, Sumaré, SP, Brazil) before recording, following the manufacturer’s instructions. The white electrode was placed on the center of the manubrium; the red electrode, on the xiphoid process; the black electrode, on the left anterior axillary line on the sixth rib; and the green electrode, on the right anterior axillary line on the sixth rib.

For time-domain analysis, the standard deviation of all NN intervals (SDNN) and the percentage of consecutive RRi with differences greater than 50 ms (pNN50%) were calculated.

The frequency domain was analyzed with the Fast Fourier Transform test for previously selected RRi sequences with 4-Hz interpolation and 50% overlap. Two main spectral components were selected for analysis: low frequency (sympathetic and parasympathetic components [LF, from 0.04 to 0.15 Hz]) and high frequency (parasympathetic component [HF, from 0.15 to 0.50 Hz]). Normalized spectral components (LF, HF, and LF/HF ratio) were expressed in normalized units (nu). Normalization consisted of dividing the power of a given spectral component (HF or LF) by the total power minus the power below 0.04 Hz and multiplying this ratio by 100.

### 2.4. Data Processing and Statistical Analyses

Data were processed and analyzed in SPSS software (SPSS Inc., Chicago, IL, USA, Release 16.0.2, 2008). Descriptive statistics were made with the Shapiro–Wilk test and Bartlett’s criteria. Continuous variables were summarized with means and standard deviations (SD), while categorical variables were presented in frequencies and percentages. Comparisons between the means of the different conditions were made with ANOVA and Tukey’s post hoc test. All statistical methods were two-tailed, *p*-value calculations were exact, and the significance level was set to 5%.

## 3. Results

### 3.1. Blood Pressure

TS and/or AAS are generally used in defined periods, often with non-use intervals. The study considered TS or AAS consumption in the 30 days before the evaluation. The mean (±SD) length of self-reported TS use by the TSG was 3.0 (±1.5) months, with a weekly frequency of 5 to 7 days. TSG reported a concomitant self-consumption of 6.0 (±1.5) grams of caffeine and 1.2 (±0.6) grams of DMAA in the preceding month. AASG reported a combination of intramuscular and oral AAS self-use with a mean of 3.6 g (±1.2) of testosterone or equivalent in the same period. Reported intramuscular AAS injections contained testosterone or derivatives, while combined oral preparations contained oxymetholone and oxandrolone. The mean length of self-reported AAS use by the AASG was 4.0 (±1.0) years. Table 1 shows statistically similar demographic characteristics (*p* > 0.05) between the groups.

Significant statistical differences (*p* < 0.05) for systolic BP (SBP) and diastolic BP (DBP) were observed between the groups in both wake and sleep periods, except for the maximum DBP during sleep (Table 2). The mean SBP during sleep was statistically different between the three groups (*p* = 0.009), with CG presenting lower mean values than the others (Δ = −13.7 mmHg in comparison with TSG [*p* < 0.01]; Δ = −23.0 mmHg in comparison with AASG [*p* < 0.01]). During sleep, the maximum SBP was higher in AASG than CG (Δ = 17.8 mmHg; *p* < 0.01), and the mean DBP was higher in TSG than CG (Δ = 8.5 mmHg; *p* < 0.01). In addition, TSG had lower values for mean SBP during sleep than AASG (Δ = −9.3 mmHg; *p* < 0.01). In the same way, maximum SBP, maximum DBP, mean SBP, and mean DBP in wake periods had lower mean values in CG than in the other groups (*p* < 0.01). The mean SBP in wake periods was statistically higher in AASG than TSG (Δ = 9.6 mmHg; *p* < 0.05).

### 3.2. Autonomic Cardiac Control

The results of ACC analysis in RT practitioners for the wake and sleep periods using time-domain methods showed higher pNN50 values in the CG during wake periods (*p* < 0.01) and in SDNN and pNN50 values during sleep periods (*p* < 0.01) in comparison with TSG and AASG (Table 3).

Likewise, frequency-domain ACC analysis showed the same trend with good CG scores (Table 4). CG had statistically lower (*p* < 0.001) LF values and LF/HF ratio during sleep and wake periods than TSG and AASG (*p* < 0.001). In addition, HF values during sleep and wake periods were statistically higher in the CG than in the other groups (*p* < 0.001). Except for HF in wake periods, all other variables had values with statistical differences in AASG in comparison with TSG (*p* < 0.05).

## 4. Discussion

The main findings of the present study are the changes in mean systolic and mean diastolic BP and ACC (SDNN; pNN50; HF; LF; LF/HF ratio) during sleep, comparing RT practitioners who do not take ergogenic aids with TS self-users and AAS self-users. In addition, this is the first study to analyze BP and ACC during sleep in this population.

These findings have important clinical and public health implications since increased SBP during sleep strongly predicts cerebrovascular disease [18,21]. Also, a decline in BP (<10%) during sleep is associated with a lower risk of morbidity and mortality from cerebrovascular disorders [22,23]. High BP during sleep may indicate persistent sympathetic hyperactivity, thus contributing to cardiovascular disease onset [21,22,23,24].

Changes in BP were also observed in the wake period for all groups. Corroborating these findings, a previous study by Gomes Gonçalves e Silva et al. [7] has demonstrated an increase in SBP in RT practitioners who took AAS or TS, in comparison with those who had never taken them. In line with the findings, research points out that supplement use (dimethylamine, alone or in combination with caffeine), can damage SBP and DBP control and mean BP [4,25,26]. In addition, AAS abuse is associated with cardiovascular impairments (cardiac arrhythmia, systemic arterial hypertension, cardiac hypertrophy, myocardial infarction) and sudden death [8,9,10,11,12,13,14,15,16,17]. 

According to Urhausen, Albers, and Kindermann [9], several years after discontinuing AAS abuse, RT athletes still have slightly concentric left ventricular hypertrophy compared to athletes who had never taken them. Additionally, chronic AAS use changes the tonus and reflex control of the cardiovascular system [10]. In this sense, cardiac receptors and BP receptors changes are essential factors to consider in cardiovascular AAS actions, according to Beutel et al. [10]. In addition to these impairments, AAS is associated with endothelial dysfunction with a consequent increase in the risk of atherosclerosis [8].

Hence, studies have shown an increase in BP with TS use and/or AAS self-medication. However, the time (duration) and/or amount per cycle necessary to cause such changes remain poorly understood. As previously mentioned, findings on long-term caffeine intake and changes in BP are conflicting.

Another important finding in the present study is the worse ACC during sleep in AASG and TSG than in CG. SDNN, pNN50, HF, LF, and LF/HF ratio values were statistically different (*p* < 0.001) between CG and the other groups during sleep. 

These findings also have important clinical implications since reduced heart rate variability during sleep is associated with the risk of stroke [27]. Evidence has demonstrated that ACC during sleep was independently associated with an increased risk of cardiovascular disease and a higher risk of lethal events after myocardial infarction [20,28]. Additionally, an elegant study by Zhang et al. [20] has demonstrated that changes in heart rate variability during sleep might occur many years before the onset of cardiovascular diseases.

Besides such damages, the present study also observed changes in ACC in wake periods in AASG, in contrast with CG and TS. Corroborating these findings, evidence has demonstrated cardiovascular impairment after AAS or TS use [4,5,6,8,9,10,11,12,13,14,15,16]. In this sense, in recent research on the effects of long-term AAS abuse on cardiac autonomic efficacy and cardiac adaptations in strength-trained athletes, impaired heart rate variability was associated with early left ventricular diastolic dysfunction [29]. Hence, impaired ACC (negative changes in the interaction between the central and peripheral nervous systems and afferent and/or efferent pathways) increases the risk of cardiac impairments.

The research design and small sample size may be regarded as limitations of this study. However, our findings encourage further studies on the topic. 

## 5. Conclusions

The findings of this study demonstrate negative changes in cardiovascular parameters during sleep and wake periods in RT after using ergogenic aids. Further randomized controlled trials with tighter control of sources of invalidation (e.g., experimental studies using randomized tests before and after treatment and evaluations conducted by blinded investigators) are needed to understand the mechanisms associated with the changes found in this research.

## Figures and Tables

**Table 1:** Comparison of demographic and anthropometric characteristics of RT practitioners who take thermogenic supplements (TS) or anabolic-androgenic steroids (AAS), or who are ergogenic aid non-users (Control).

Variables	Groups			*p* ^#^
	Control (*n* = 15)	TS (*n* = 15)	AAS (*n* = 15)	
Age, years	24.7 ± 5.4	24.8 ± 3.7	21.7 ± 3.3	0.086
Height, m	1.77 ± 0.05	1.76 ± 0.07	1.74 ± 0.07	0.433
Weight, kg	82.4 ± 11.4	80.3 ± 9.3	73.9 ± 12.4	0.105
Body mass index, kg·m^2^	26.2 ± 3.5	26.1 ± 3.4	24.2 ± 3.1	0.899
Testosterone, g	-	-	3.6 ± 1.2	-
Caffeine, g	-	6.0 ± 1.5	-	-
Dimethylamylamine, g	-	1.2 ± 0.6	-	-

TS: thermogenic supplements; AAS: anabolic-androgenic steroids; m: meter; kg: kilogram; g: gram. ^#^ *p*-values obtained in one-way ANOVA.

**Table 2:** Comparison of blood pressure levels in sleep and wake periods between RT practitioners who take thermogenic supplements (TS) or anabolic-androgenic steroids (AAS), or who are ergogenic aid non-users (Control).

Evaluation Moments	Groups			*p* ^#^
	Control	TS	AAS	
	(*n* = 15)	(*n* = 15)	(*n* = 15)	
Sleep				
Maximum SBP, mmHg	119.5 ± 6.5 ^A^	128.5 ± 14.9	137.3 ± 17.7 ^B^	0.004
Maximum DBP, mmHg	63.3 ± 12.2	67.9 ± 11.0	66.4 ± 12.5	0.564
Mean SBP, mmHg	103.6 ± 4.0 ^A^	117.3 ± 12.3 ^B^	126.6 ± 12.4 ^C^	<0.001
Mean DBP, mmHg	55.7 ± 7.6 ^A^	64.2 ± 6.5 ^B^	61.5 ± 7.9	0.009
Wake				
Maximum SBP, mmHg	126.9 ± 10.2 ^A^	133.5 ± 11.8 ^B^	150.5 ± 15.0 ^B^	<0.001
Maximum DBP, mmHg	70.2 ± 10.3 ^A^	73.9 ± 8.1 ^B^	93.8 ± 24.7 ^B^	<0.001
Mean SBP, mmHg	117.4 ± 5.8 ^A^	127.4 ± 11.3 ^B^	137.0 ± 10.6 ^C^	<0.001
Mean DBP, mmHg	64.1 ± 7.1 ^A^	71.2 ± 6.6 ^B^	72.5 ± 7.2 ^B^	0.004

TS: thermogenic supplements; AAS: anabolic-androgenic steroids; SBP: systolic blood pressure; DBP: diastolic blood pressure; mmHg: millimeter of mercury. ^#^ *p*-values were obtained with one-way ANOVA, and the different letters represent statistical differences (*p* < 0.05) in the post hoc test. A: *p* < 0.05 in comparison with B and/or C; B: *p* < 0.05 in comparison with C.

**Table 3:** Comparison of time-domain analysis of heart rate variability in sleep and wake periods between RT practitioners who take thermogenic supplements (TS), anabolic-androgenic steroids (AAS), or who are ergogenic aid non-users (Control).

Evaluation Moments	Groups			*p* ^#^
	Control	TS	AAS	
	(*n* = 15)	(*n* = 15)	(*n* = 15)	
Sleep				
SDNN, ms	157.7 ± 31.5 ^A^	121.2 ± 29.7 ^B^	117.6 ± 22.2 ^B^	<0.001
pNN50, %	51.1 ± 17.8 ^A^	30.5 ± 16.7 ^B^	31.6 ± 15.6 ^B^	0.002
Wake				
SDNN, ms	138.3 ± 53.6	96.9 ± 28.5	109.3 ± 54.8	0.059
pNN50, %	23.9 ± 11.0 ^A^	12.8 ± 13.2 ^B^	9.7 ± 9.7 ^B^	0.004

TS: thermogenic supplements; AAS: anabolic-androgenic steroids; SDNN: standard deviation of NN intervals; pNN50: percentage of successive RR intervals with more than a 50-millisecond difference; ms: millisecond. ^#^ *p*-values were obtained with one-way ANOVA, and the different letters represent statistical differences (*p* < 0.05) in the post hoc test. A: *p* < 0.05 in comparison with B.

**Table 4:** Comparison of the frequency-domain analysis of heart rate variability in sleep and wake periods between RT practitioners who take thermogenic supplements (TS) or anabolic-androgenic steroids (AAS), or who are ergogenic aid non-users (Control).

Evaluation Moments	Groups			*p* ^#^
	Control	TS	AAS	
	(*n* = 15)	(*n* = 15)	(*n* = 15)	
Sleep				
LF, nu	46.0 ± 10.2 ^A^	66.6 ± 12.1 ^B^	79.0 ± 15.4 ^C^	<0.001
HF, nu	45.9 ± 6.0 ^A^	36.2 ± 7.0 ^B^	29.6 ± 6.3 ^C^	<0.001
LF/HF ratio	1.0 ± 0.2 ^A^	1.9 ± 0.5 ^B^	2.6 ± 0.9 ^C^	<0.001
Wake				
LF, nu	42.8 ± 9.8 ^A^	69.2 ± 9.3 ^B^	80.5 ± 4.4 ^C^	<0.001
HF, nu	42.9 ± 5.5 ^A^	31.2 ± 8.7 ^B^	25.8 ± 5.7 ^B^	<0.001
LF/HF ratio	1.0 ± 0.3 ^A^	2.4 ± 0.8 ^B^	3.4 ± 1.1 ^C^	<0.001

TS: thermogenic supplements; AAS: anabolic-androgenic steroids; LF: low frequency; HF: high frequency; nu: normalized unit. ^#^ *p*-values were obtained with one-way ANOVA, and the different letters represent statistical differences (*p* < 0.05) in the post hoc test. A: *p* < 0.05 in comparison with B and/or C; B: *p* < 0.05 in comparison with A and/or C; C: *p* < 0.05 in comparison with A and/or B (unnecessary by the explanation for A or B).

## Data Availability

Not applicable.
